# Characterizing microglia activation: a spatial statistics approach to maximize information extraction

**DOI:** 10.1038/s41598-017-01747-8

**Published:** 2017-05-08

**Authors:** Benjamin M. Davis, Manual Salinas-Navarro, M. Francesca Cordeiro, Lieve Moons, Lies De Groef

**Affiliations:** 1 Glaucoma and Retinal Neurodegenerative Disease Research Group, Institute of Ophthalmology, University College London, 11-43 Bath Street, London, EC1V 9EL United Kingdom; 2 Neural Circuit Development and Regeneration Research Group, Department of Biology, University of Leuven, Naamsestraat 61 box 2464, 3000 Leuven, Belgium; 3 Western Eye Hospital, Imperial College Healthcare NHS Trust, 171 Marylebone Road, London, NW1 5QH United Kingdom

**Keywords:** Cellular imaging, Neurodegeneration, Microglia, Retina

## Abstract

Microglia play an important role in the pathology of CNS disorders, however, there remains significant uncertainty about the neuroprotective/degenerative role of these cells due to a lack of techniques to adequately assess their complex behaviour in response to injury. Advancing microscopy techniques, transgenic lines and well-characterized molecular markers, have made histological assessment of microglia populations more accessible. However, there is a distinct lack of tools to adequately extract information from these images to fully characterise microglia behaviour. This, combined with growing economic pressures and the ethical need to minimise the use of laboratory animals, led us to develop tools to maximise the amount of information obtained. This study describes a novel approach, combining image analysis with spatial statistical techniques. In addition to monitoring morphological parameters and global changes in microglia density, nearest neighbour distance, and regularity index, we used cluster analyses based on changes in soma size and roundness to yield novel insights into the behaviour of different microglia phenotypes in a murine optic nerve injury model. These methods should be considered a generic tool to quantitatively assess microglia activation, to profile phenotypic changes into microglia subpopulations, and to map spatial distributions in virtually every CNS region and disease state.

## Introduction

Whilst small in size, microglia are of enormous importance for CNS health and repair. Fulfilling their neuroinflammatory role, these so-called ‘resident macrophages’ continuously probe the CNS environment for signs of distress, and migrate to sites of injury/infection/neurodegeneration where they recruit and activate additional cells, proliferate, phagocytose, clear debris and reorganize the CNS parenchyma. This microglial reactivity is a complex multistage activation process, which broadly encompasses their transformation from a ramified to an amoeboid morphology by first retracting the microglia processes and then extending dynamic protrusions, followed by cellular locomotion. Microglia dynamics are, however, sometimes very subtle, and go beyond their immune surveillance function. Indeed, microglia are believed to represent an important nexus between neurological and immunological activity in the CNS, and, for example, are also essential for synaptic homeostasis and regulation of neurotransmitters^1–3^.

Despite their central role in mounting a rapid, protective response against perturbations of CNS homeostasis, microglial activity has also been linked to various pathological conditions including neurodegenerative diseases. Indeed, while microglial reactivity may at first aid to the elimination of the source of distress and to the restoration of tissue homeostasis, persistent inflammatory stimuli (as present in e.g. Alzheimer’s, Parkinson’s diseases, amyotrophic lateral sclerosis) may ultimately overwhelm microglia and lead to a downward spiral of chronic inflammation that compromises neuronal survival^4, 5^. Altogether, in response to their ever-changing surroundings, microglia display a remarkable degree of phenotypic plasticity – both in terms of morphology, number and molecular markers – and may be skewed to either a neurotoxic or a neuroprotective role. Much remains to be investigated, however, in order to understand this dynamic process and to correlate microglia phenotypes and function.

Historically, microglia phenotypes have been described by categorizing cells based on their morphological features. In general, highly ramified cells were designated ‘resting’ microglia and amoeboid cells were called ‘activated’, with a range of intermediate activation states in between (e.g., ‘intermediately activated’, ‘bipolar’, ‘rod-like’, ‘hypertrophied’, ‘bushy’). No morphological classification standard exists, however, which complicates comparison of data from different studies, and prevents objective quantification of pathological status and therapeutic effects. Nevertheless, it is well-known that microglia morphological features and function are tightly coupled^3, 6^, and the development of unbiased and quantitative methods could therefore lead to improved insights into this form-function paradigm. Furthermore, microglia reactivity is a highly dynamic process, which is likely to be better described by a continuum of quantitative physiological measures – able to detect subtle changes in cell morphology and to capture the dynamics of microglia plasticity – rather than by a qualitative categorization^3, 7^. Over the last years, novel methodological approaches for adequate description of the reactive transformation of microglia have been emerging (e.g., refs 3, 8–12), yet correlating microglia morphology to function remains difficult and few easy-accessible user-friendly tools are presently available to adequately quantify morphological changes. In addition, microglia phenotypes appear to heterogeneous, both amongst CNS regions and within a single tissue, which emphasizes the need for comprehensive analysis of the entire microglia population.

In the current study, microglial reactivity was evaluated on whole-mounted mouse retinas, harvested four days after ONC. The ONC model has been extensively used to study retinal neurodegeneration, and the timing and pathological mechanisms leading to retinal ganglion cell death have been the subject of many studies, e.g. refs 13–16. Furthermore, a growing interest in the detailed course of microglial reactivity in this experimental glaucoma model has been emerging^17–21^. Aiming to address this recent focus, we designed an automated analysis algorithm that, based on Iba-1 (ionized calcium-binding adapter molecule 1) immunostaining of the retinal microglia, quantifies five parameters: cell density, nearest neighbour distance (NND) and regularity index, to describe microglia numbers and distribution; and cell soma size and roundness, to assess cell morphology. Together, in combination with a spatial statistics approach, this comprehensive set of measures allows us to extract greater information from whole-retinal histology than has previously been possible, to quantitatively describe the dynamic pathological state of microglia in the retina.

## Results

### Quantification of microglial activation by a novel comprehensive image analysis script

**Figure 1:**
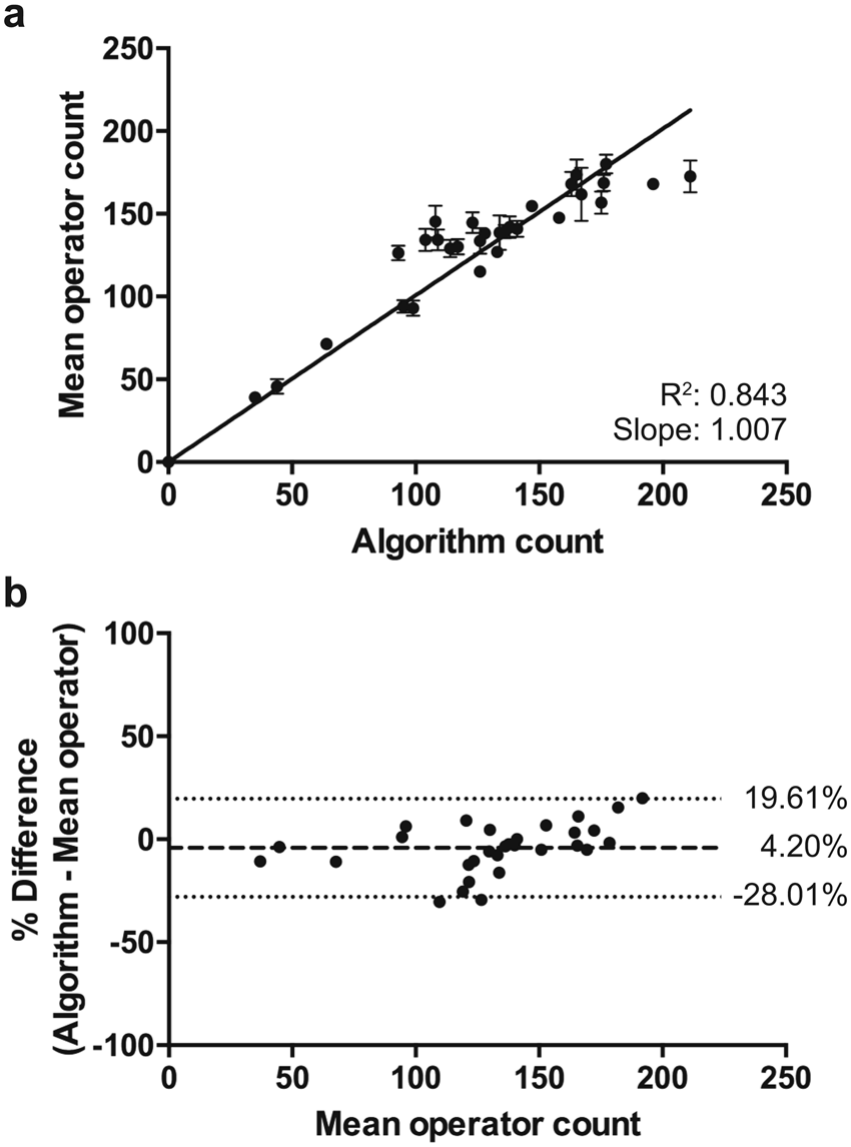
Algorithm optimization. (**a**) Linear fit of the mean operator counts *versus* the numbers of microglia detected by the automated algorithm, reveals a strong correlation (y = 1.007x; R^2^ = 0.843) and validates our algorithm. (**b**) This is confirmed by the Bland-Altman plot, which shows a bias of 4.20%. For comparison, bias for three observers was −8.35%, 4.33% and 3.50%. Data are depicted as mean ± 95% confidence intervals.

Retinal microglia were visualized by means of immunohistochemical staining for Iba-1, a commonly used macrophage/microglia-specific calcium binding protein and well-suited marker for morphological differentiation of ramified *versus* amoeboid microglia^22^. Automated detection and counting of Iba-1 immunopositive cells was validated by comparison to manual counts by three masked observers. Algorithm results correlated strongly with mean manual observations and subsequent Bland-Altman analysis^23^ revealed comparable inter-operator variability between manual observers and the algorithm described herein (Fig. 1, Table 1).

**Table 1:** Correlation analysis of automated detection *versus* manual counting of Iba-1 immunopositive cells.

	Slope	R2	Pearson R	p-value	Bias (%)	95% CI Upper	95% CI Lower
Observer 1	1.072	0.920	0.9787	<0.0001	−8.347	6.626	−23.32
Observer 2	0.963	0.873	0.9479	<0.0001	4.328	21.77	−13.12
Observer 3	0.956	0.885	0.9620	<0.0001	3.499	−14.09	21.09
Algorithm	1.007	0.843	0.9376	<0.0001	4.203	19.61	−28.01

### Analysis of cell number and distribution to measure microglial reactivity

**Figure 2:**
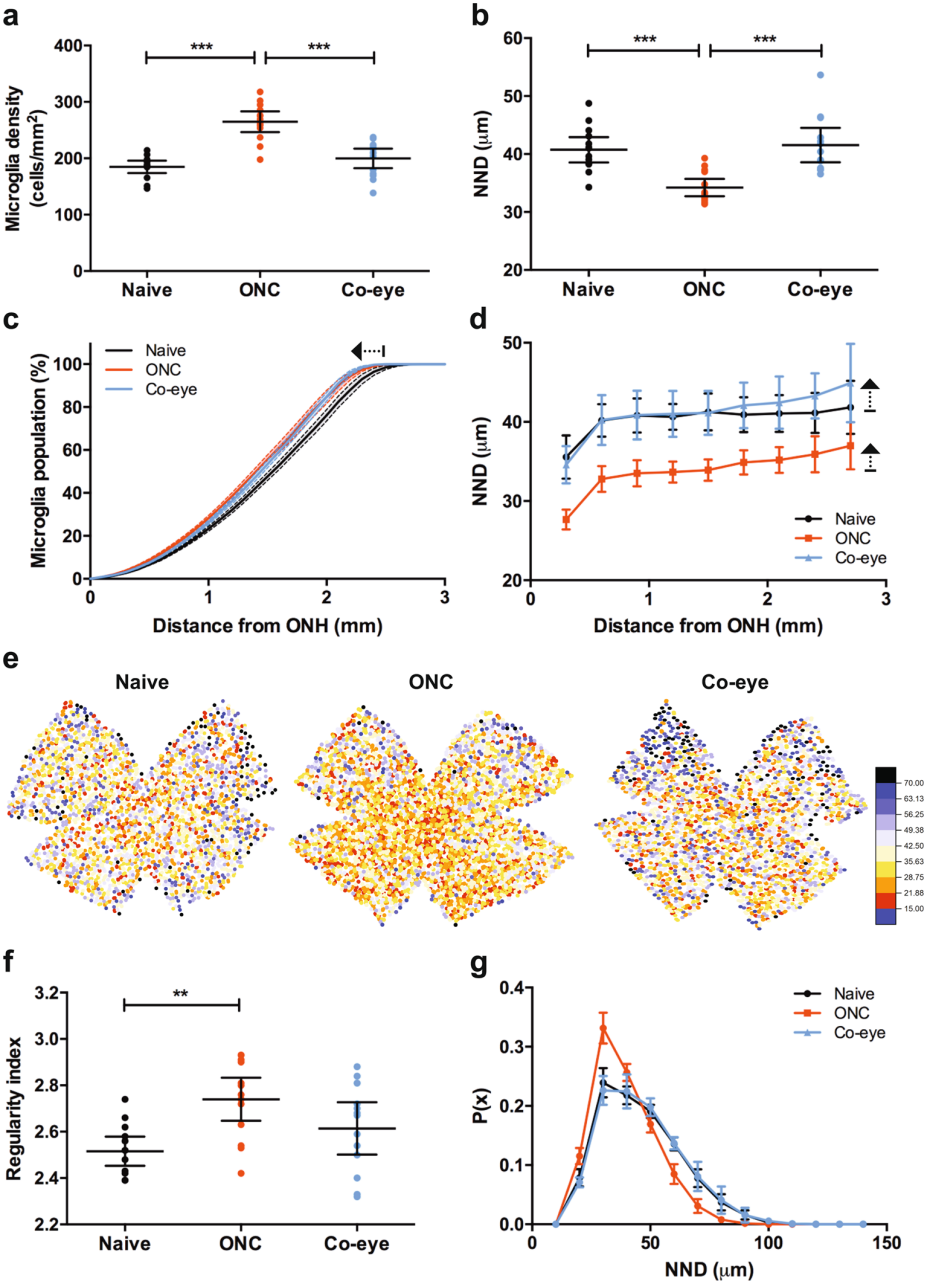
Microglia numbers and distribution. (**a**) A significant increase in overall microglia density is seen when comparing naive *versus* ONC retinas (4 days post injury). The microglia density of microglia is not significantly different between naive retinas and retinas from the contralateral eye of the ONC animals (one-way ANOVA with Tukey’s *post hoc* test). (**b**) Nearest neighbour distance (NDD) is significantly higher in ONC retinas, but not in the contralateral eye (one-way ANOVA with Tukey’s *post hoc* test). (**c**) Plotting cumulative microglia density, depicted as a percentage of the total microglia number, reveals that retinal microglia density is higher in the central regions of retina in ONC and contralateral eyes *versus* naive eyes. This can be observed as a left-shift of the ONC and Co-eye curves (arrow). (**d**) Distribution analysis of NND, in relation to the distance from the ONH, indicates that overall NND decreases in ONC eyes in comparison to naive eyes. However, NND disproportionally increases in the peripheral retina, a pattern that can also be discerned in the retinas of contralateral eyes (arrows). (**e**) Pseudocolor images of microglia NND/density in retinal whole-mounts. Red-to-blue pseudocolour representation of microglia distribution, ranging from small NND (15 μm, red tones) to large NND (70 μm, blue tones). (**f**) The regularity index of the microglia population increases upon ONC (one-way ANOVA with Tukey’s *post hoc* test). (**g**) A probability function of the NND points out that both the mean NND and NND standard deviation decrease after ONC, which explains the increase in regularity index. Data are depicted as mean ± 95% confidence intervals.

The first set of parameters that was analysed in our data set comprised cell density, NND and regularity index, and aimed to assess microglia numbers and distribution. First, upon comparison of microglia cell numbers in retinas of naive mice *versus* retinas that underwent ONC (4 days post injury), overall cell density was found to dramatically increase by 43% (185 ± 5 cells/mm^2^ *versus* 264 ± 9 cells/mm^2^, respectively; P < 0.005) (Fig. 2a). This was also reflected in the NND, which decreased in the ONC retinas (40.8 ± 1.0 μm *versus* 34.2 ± 0.7 μm; P < 0.005) (Fig. 2b). A detailed analysis of microglia density and NND in function of the distance from the ONH, furthermore revealed that microglia density tends to be higher in the central retina for all treatment groups (Fig. 2c,d). In addition, the left-shift in the cumulative density graphs (Fig. 2c, arrow) and the larger NND at increasing distance from the ONH (Fig. 2d, arrows), are suggestive of a microglia redistribution from the periphery to the centre in response to the ONC injury. Notably, this microglial response appeared to be almost identical in ONC and contralateral eyes at 4 days post ONC, and – given that the overall microglia density did not change in the contralateral eyes – proposes that at least part of the increased central cell density in the ONC retinas is the result of relocating resident microglia. These spatial organizations are illustrated by the pseudocolour maps, giving a graphical representation of microglia NND (Fig. 2e).

Second, the regularity index, which is defined as the ratio of the mean NND over the NND standard deviation and describes the regular spacing of cells, was seen to increase upon ONC (2.52 ± 0.03 *versus* 2.74 ± 0.04; P < 0.01) (Fig. 2e). Indeed, looking at the probability function of the NND, one can see that the mean NND shifts to the left (*i.e*., becomes smaller) and the distribution becomes more narrow (*i.e*., the standard deviation decreases) upon ONC (Fig. 2f). In other words, the NND in a population of activated microglia becomes smaller and more homogenous as the cells are more densely packed and their exclusion radii touch. The latter will be further explored in the following sections with Ripley’s *K*-statistics.

Intriguingly, analysis of microglia density, NND and regularity index in the contralateral eye of animals that underwent ONC, revealed that values tended to deviate from the naive eye. Although these differences were small and found not to be significant in this study, they are suggestive of a mild microglial activation in the contralateral eye in response to ONC (Fig. 2a–e).

### Analysis of cell shape to measure microglial reactivity

**Figure 3:**
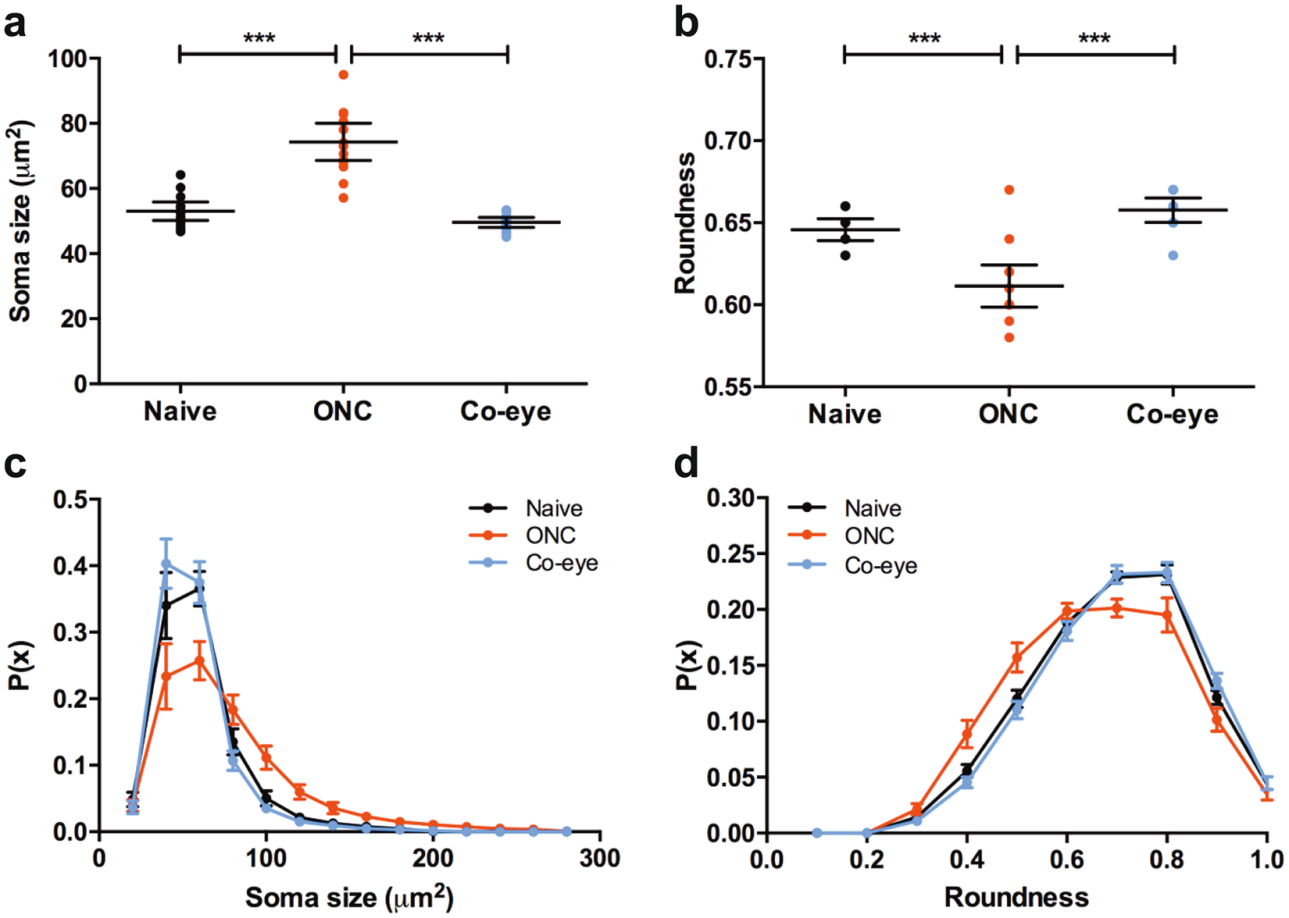
Microglia soma size and roundness. (**a**) Microglial activity following ONC is associated with an increase in average cell body area; while (**b**) roundness of the cell soma decreases (one-way ANOVA with Tukey’s *post hoc* test). These signs of microglial activation are not seen in the retina of the contralateral ONC eyes. (**c**) Distribution analysis of soma area shows a shift from small to larger cell body sizes after ONC; and (**d**) distribution analysis of circularity indicates that these cells are more likely to be more irregularly shaped (*i.e*., to have a lower roundness index). Data are depicted as mean ± 95% confidence intervals.

A more detailed analysis focusing on the microglia cell somata, revealed two additional parameters that were significantly affected upon ONC. First, compared to naive mice, average cell body size increased (53.1 ± 1.3 μm^2^ *versus* 74.3 ± 2.7 μm^2^; P < 0.005) (Fig. 3a). Second, microglial reactivity at 4 days post ONC was also characterized by a reduction in the roundness of the cell body (0.181 ± 0.006 *versus* 0.160 ± 0.006 in naive and ONC retinas, respectively; P < 0.05) (Fig. 3b). Distribution analyses furthermore revealed a wider, slightly right-shifted distribution for soma size and left-shifted distribution for roundness (Fig. 3c,d). Together, these data suggest that the number of microglia with a small, round cell body decreased after ONC and that these cells adopted a bigger, more irregular soma shape. This fits the general classification of microglia morphology, which describes the surveying ‘resting’ cells as small, round cells with elaborate ramifications, and the ‘activated’ microglia as bigger, more amoeboid cells with retracted processes. Of note, no signs of activation were found when analysing cell soma size and roundness in the retina of the contralateral eye of the ONC animals (Fig. 3a–d).

### *K*-means clustering to gain a deeper insight into microglia subpopulations with different activation states

**Figure 4:**
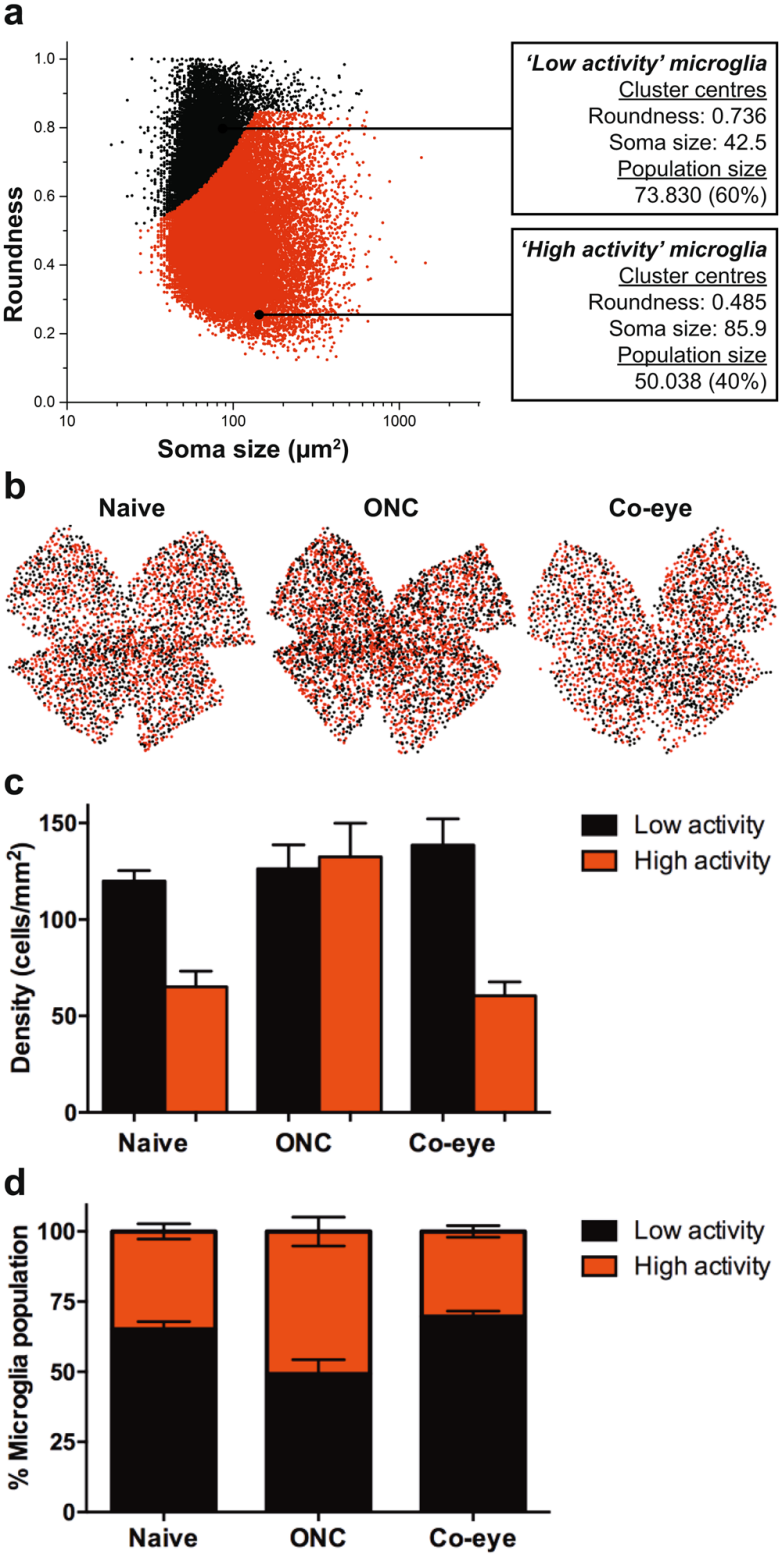
*K*-means clustering into ‘low activity’ and ‘high activity’ microglia subpopulations. (**a**) Two subpopulations of microglia were defined via *K*-means cluster analysis of a total of 123.868 cells (*i.e*., the sum of microglia from 14 naive retinas, 14 ONC retinas and 14 contralateral retinas), based on their roundness and soma size. (**b**) Images of representative retinal whole-mounts from naive, ONC and contralateral eyes, showing the distribution of ‘high activity’ (red) and ‘low activity’ (black) microglia. Each dot represents a microglia cell. (**c**) Absolute numbers of ‘low activity’ and ‘high activity’ microglia in the retina reveal that microgliosis after ONC merely presents as an increase in the number of ‘high activity’ microglia (one-way ANOVA with Tukey’s *post hoc* test). (**d**) This results in an increased proportion of ‘high activity’ *versus* ‘low activity’ microglia at day 4 post ONC, in comparison to naive retinas. No significant changes were observed in the contralateral eye. Data are depicted as mean ± 95% confidence intervals.

While the above measures provide an average value for microglia soma size and roundness for each retina, they fail to take into account the heterogeneity of microglia phenotypes within a single retina. Therefore, in order to define subpopulations of microglia with a ‘low’ *versus* ‘high’ activation status, we performed *K*-means clustering that divided the data into two main categories based on microglia soma size and roundness (Fig. 4a). We defined these clusters as ‘low activity’ and ‘high activity’ microglia, rather than ‘resting’ *versus* ‘activated’ cells, as we believe that this traditional classification fails to capture the diverse functions of microglia in both healthy and diseased CNS tissues. Microglia falling in the first category had small yet round somata and therefore resemble the typical morphological features of so-called resting microglia; microglia in the ‘high activity’ cluster displayed typical signs of microglial reactivity, *i.e*., an increased soma size and more irregular shape of their cell body, and thereby come close to the amoeboid-like morphology that is typically associated with activated microglia. This *K*-means clustering revealed that in a naive retina on average 65 ± 1% of the microglia fell into the ‘low activity’ and 35 ± 1% in the ‘high activity’ cluster (120 ± 3 *versus* 65 ± 4 cells/mm^2^, respectively). While the number of ‘low activity’ cells remained the same (126 ± 6 cells/mm^2^), the number of ‘high activity’ microglia doubled to 139 ± 8 cells/mm^2^ (P < 0.005) in the ONC eyes, resulting in a ratio of 49 ± 2% ‘low activity’ *versus* 51 ± 2% ‘high activity’ microglia (Fig. 4c,d). In the contralateral eye, no significant differences were seen, although the population of ‘low activity’ microglia tended to somewhat increase in comparison to naive retinas (from 65 ± 1% to 70 ± 1%) (Fig. 4c,d). Finally, graphical representations of the distribution of ‘low activity’ and ‘high activity’ microglia hint that there were no regional differences in microglia activation (Fig. 4c).

### Ripley’s *K*-statistics and Dixon’s χ^2^-test to resolve microglia territories and spatial dynamics

**Figure 5:**
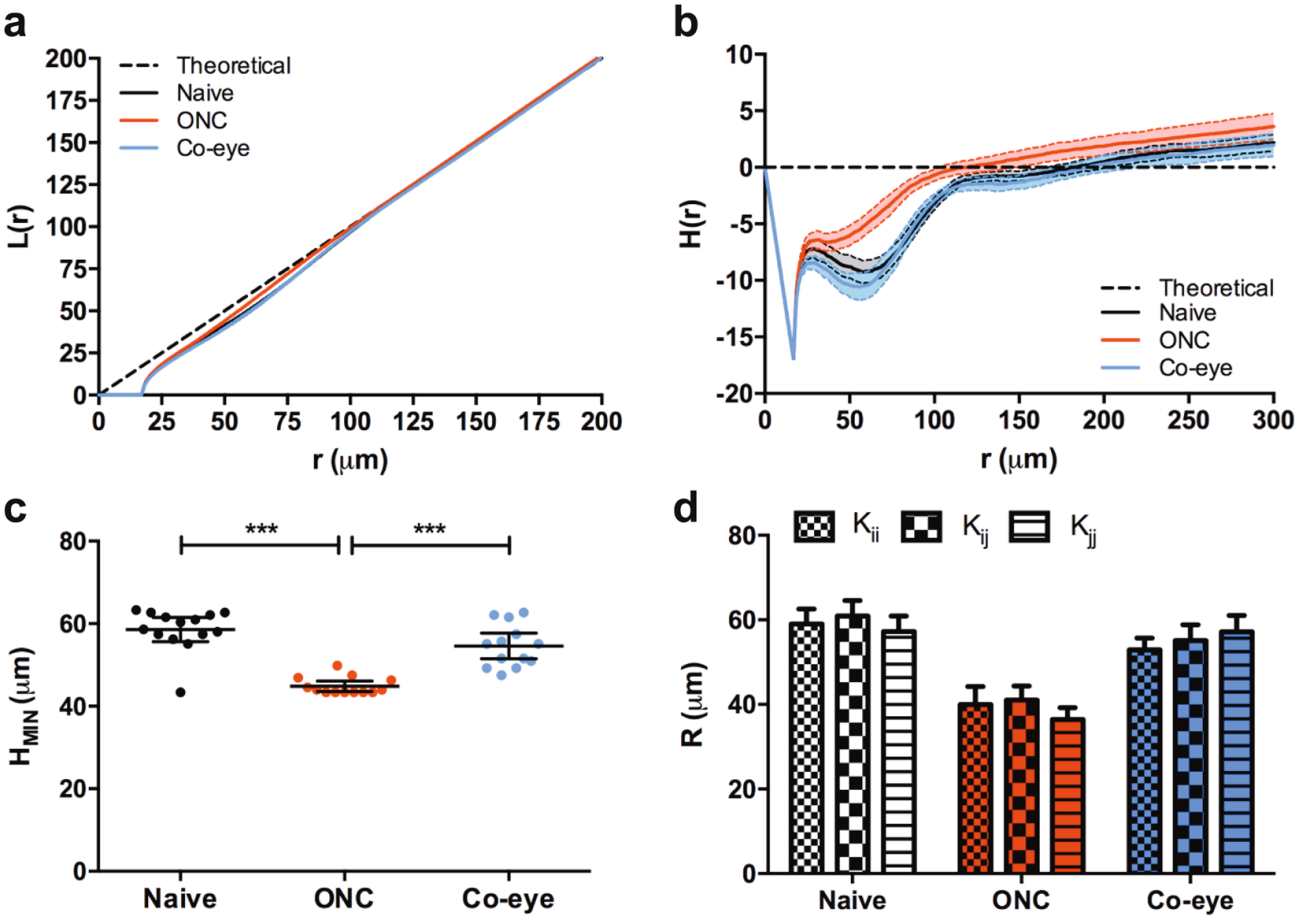
Ripley’s *K*-statistics, analysing spatial clustering of microglia subtypes. (**a**) For all treatment groups, *L*(*r*) and *H*(*r*) derivatives of Ripley’s *K*-function are negative for small values of *r*, indicating that retinal microglia have a dispersed topographical organisation. (**b**) Upon ONC, defined as the minimum of *H*(*r*), the domain radius of microglia decreases in size (one-way ANOVA with Tukey’s *post hoc* test). (**c**) Comparison of cross-*K* (*K*_*ij*_) and self-*K* functions (*K*_*ii*_ and *K*_*jj*_) shows that ‘low activity’ and ‘high activity’ microglia subpopulations evenly decrease their domain radius after ONC injury (two-way ANOVA with Tukey’s *post hoc* test). Data are depicted as mean ± 95% confidence intervals.

In a spatial statistics analysis, using *L*(*r*) and *H*(*r*) derivatives of Ripley’s *K*-function, we investigated the topographical organization of retinal microglia. The negative values found for *L*(*r*) and *H*(*r*) indicate that microglia tend to disperse rather than cluster together in foci (Fig. 5a,b). Calculating the second minimum of the derivative of *H*(*r*) yielded the average size of the microglia territories under the different experimental conditions (Fig. 5b). In naive and ONC eyes, the average microglia domain radius was 58.6 ± 1.4 μm and 44.9 ± 0.6 μm, respectively, indicating that microglia activation coincides with a reduction in size of the microglia territories (P < 0.005) (Fig. 5c). Notably, in the contralateral eyes, microglia also somewhat decreased their domain radius (54.6 ± 1.5 μm), yet this effect was not significant. Running cross-*K* (*K*_*ij*_) and self-*K* functions (*K*_*ii*_ and *K*_*jj*_) (where *i* stands for ‘low activity’ microglia and *j* for ‘high activity’ microglia) furthermore revealed that, in response to ONC, both ‘low activity’ and ‘high activity’ microglia reduce their domain radii to a similar extent (Fig. 5d). Domain radii are thus modulated independent of activation status.

Finally, Dixon’s χ^2^-test for testing spatial segregation was applied, focusing on the identity (*i.e*., ‘low activity’ or ‘high activity’) of the nearest neighbour of each microglia rather than the distance between them. A Dixon’s χ^2^-test was applied to the distribution of ‘low activity’ or ‘high activity’ microglia *versus* simulated random distributions, to examine whether microglia had a tendency to cluster beyond the exclusion radii identified above. A summary of the number of ‘low activity’ and ‘high activity’ microglia and the χ^2^-statistics, degrees of freedom, and p-values is provided in Table 2. The complete results are available as a Supplementary Table. Investigation of these test statistics revealed that microglia from both subpopulations have a mild tendency towards self-association. Upon ONC, however, a large increase in ‘high activity’ microglia clustering was observed. Indeed, across the 14 naive retinas included in this analysis, the aggregate C = 47.81 with 28 degrees of freedom yielded a p-value of 0.0112. Dixon’s χ^2^-test thus points to a tendency for ‘low activity’ and ‘high activity’ microglia to spatially segregate differently that what is expected under random labelling. The p-values of squared-summed *z*_*ii*_ = 25.00 (*P* = 0.0346) and *z*_*jj*_ = 26.55 (*P* = 0.0220) with 14 degrees of freedom indicated that both microglia subtypes tend to self-associate. At four days post ONC, the aggregate C = 143.57 with 28 degrees of freedom had a p-value < 0.000001, indicative of a very strong deviation from random labelling and from the naive condition. The square-summed *z*_*ii*_ = 23.72 with 14 degrees of freedom and p-value = 0.0495 corresponded to our observations in the naive retina, suggesting that ‘low activity’ microglia did not behave differently in naive *versus* ONC retinas. In contrast, the squared summed *z*_*jj*_ = 104.80 with 14 degrees of freedom and *P* < 000001 proposes that a large increase in ‘high activity’ microglia clustering is responsible for the increase in aggregate C *versus* naive retina. Spatial segregation of retinal microglia in the contralateral eye was similar to the naive retina. In summary, ‘high activity’ microglia preferentially self-associate, which could be explained by their cytokine-mediated cross-talk leading to activation of neighbouring cells and/or recruitment of additional microglia/macrophages.

**Table 2:** Summary of Dixon’s χ^2^-test results across retinas from naive, ONC and contralateral eyes, each with *N*_*i*_ ‘low activity’ microglia and *N*_*j*_ ‘high activity’ microglia.

Condition	*N*_*i*_	*N*_*j*_	Overall			Group-specific					
			Σ*C*	df	p-value	${{\boldsymbol{\sum }}{{\bf{z}}}_{{\boldsymbol{i}}{\boldsymbol{i}}}^{{\bf{2}}}}^{}$	*df*	*P*	${{\boldsymbol{\sum }}{{\bf{z}}}_{{\boldsymbol{j}}{\boldsymbol{j}}}^{{\bf{2}}}}^{}$	df	p-value
Naive	25208	13713	47.81	28	0.0112	25.00	14	0.0346	26.55	14	0.0220
ONC	24242	25631	143.57	28	0.000001	23.72	14	0.0495	104.80	14	0.00001
Co-eye	24380	10694	49.33	26	0.0038	24.77	13	0.0248	15.09	13	0.3017

## Discussion

Given that changes in microglia activation can be elucidated from changes in population size and morphology, independent of any molecular markers of activation, quantitative analysis these parameters can be used to assess the pathologic state of a given tissue. In this study, we exemplified this approach by describing microglia activation in a mouse ONC model, based on a five-parameter morphological analysis. Initially, we performed a global analysis of these activity markers in the entire retina and calculated average values, using traditional parametric statistical methods. Next, non-parametric cluster analysis and spatial segregation statistics were applied to this data set, in order to investigate the heterogeneity of microglia subpopulations and their spatial organization within the retina.

### A comprehensive view on retinal microglia activation following optic nerve injury

**Figure 6:**
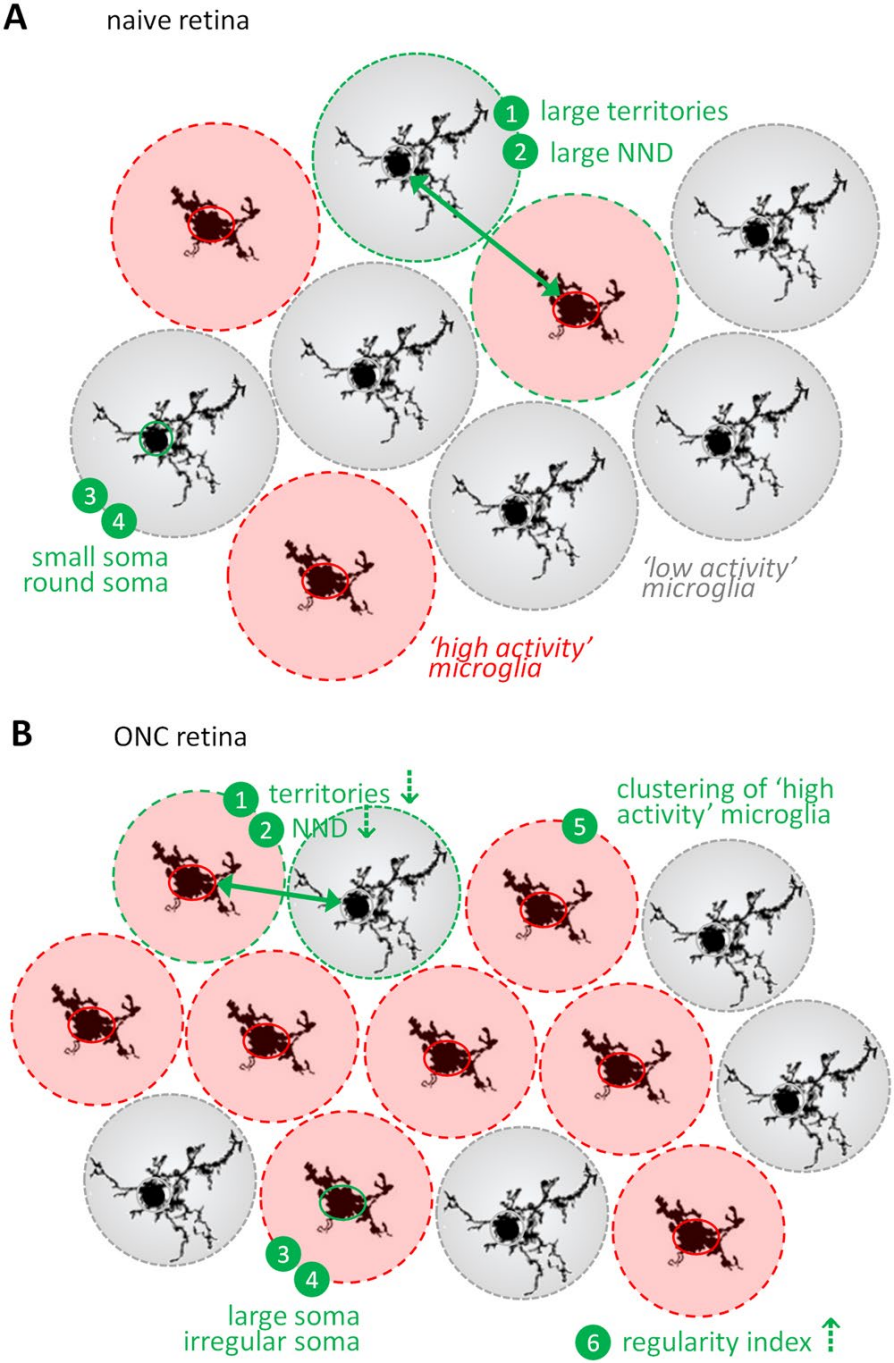
Summary figure integrating all findings from this study. (**a**) In a naive retina, microglia typically have small somata and large territories, corresponding to a ‘low activity’ status (grey). They are regularly interspersed with a minority of ‘high activity’ cells (red). (**b**) At day 4 post ONC, microglia density has increased and the majority of the microglia has adopted a ‘high activity’ phenotype, characterized by a reduction in their territory, enlargement of their cell body and a more irregular cell shape. Furthermore, although ‘low activity’ and ‘high activity’ microglia are regularly spaced, ‘high activity’ microglia tend to self-associate.

Together, the results from this study can be integrated in order to model how microglia number, distribution, and morphology change in relation to microglia activation (Fig. 6). This shows that, at four days post ONC, microglial activation in the retina leads to, first, microglia proliferation/macrophage infiltration, which is visible as an increase in the density of Iba-1^+^ cells; and, second, to a more closely packed yet regularly spaced organization, as evidenced by the measures of nearest neighbour distance and regularity index. More in-depth analysis of microglia topographic organization via Ripley’s *K*-statistics confirms that their exclusion radius reduces upon ONC. This plasticity of microglia territories appears to be conserved in all treatment groups, suggesting that this may constitute an intrinsic cell-cell interaction mechanism that ensures efficient microglia surveillance covering the entire retina. As the radii of these territories were found to be similar for ‘low activity’ and ‘high activity’ microglia, this phenomenon appears to be independent from microglia size/morphology (*i.e*., the territory covered by microglia processes), but may instead be the result of chemotactic signalling^24^. In addition, microglia activation is associated with an increase in cell body size and a decrease in its roundness, reflecting the morphological transition from a ramified ‘resting’ to an activated amoeboid-like phenotype. Based on these two measures, we performed a *K*-means clustering to segment microglia populations into two subtypes, *i.e*., ‘high activity’ and ‘low activity’ microglia. Although this clustering is somewhat artificial, by categorizing microglia into either of the two limits of their spectra of phenotypes, it can be used to gain more insight into the diverging behaviour of ‘high activity’ and ‘low activity’ microglia, provided that the limitations of this approach are considered. Indeed, re-evaluation of microglia density for each of the clusters pointed out that the increase in microglia density after ONC is merely an increase in the number of ‘high activity’ microglia. Finally, Dixon’s χ^2^ spatial statistics disclosed that these ‘high activity’ microglia are more often surrounded by other ‘high activity’ microglia, which might be a reflection of their cell-cell communication that is designed to mount a self-propagating inflammatory response (Fig. 6).

Overall, these findings agree with other studies of microglial activity in mouse ONC models of glaucomatous retinal neurodegeneration, which revealed proliferation of microglia taking place within the first week post ONC, as well as shape simplification and enlargement of their soma size^20, 21^. Despite following a different time course, identical changes in microglia appearance are also seen in other experimental rodent glaucoma models^17–19, 21, 25–30^. Notably, although not statistically significant, our findings hinted towards a low-grade immune response in the contralateral eyes, thereby corroborating previous findings in rodent glaucoma models^30–32^.

### Microscopic spatial statistics: a novel, generic approach to study microglia dynamics

The major strength of our approach, wherein its innovation lies in comparison to the traditional morphological categorization of microglia, is that it allows for the detection of changes in cell morphology in a very large number of cells. Indeed, rather than focusing on a detailed, manual/qualitative/semi-quantitative analysis of a limited number of cells in a small region of interest, we performed a population analysis including all microglia in the retina. As such, and by focusing on cell soma shape and size rather than on skeleton analysis, we choose high-throughput and moderate computing over detailed, single-cell analysis and avoid the need for high-resolution, high-magnification images. This overall approach is in contrast to fractal and Sholl analyses, the two most commonly used alternative analysis methods^3, 10, 33, 34^. Our results prove that this strategy allows to gain advanced insights into microglia activation dynamics, especially by combining conventional statistics on population averages with clustering analyses of these large data sets. Because of the very large sample sizes, combined with *K*-means, Ripley’s *K*-statistics and Dixon’s χ^2^ spatial statistics, our method is able to capture the heterogeneous morphological transformations that go with microglia activation dynamics and may – in a future approach – aid to link the continuum of these morphological subpopulations to physiological differences. Indeed, as was also recently suggested by Verdonck *et al*.^12^ – who used a similar *K*-means approach to analyse microgliosis in the brain following endotoxemia – expanding the number of parameters in the clustering analysis (e.g., additional morphological features, gene expression signatures,…) could refine the classification of microglia subtypes and lead to a better understanding of morphology-function relations. Furthermore, microscopic spatial statistical approaches can be used to shed light on how the spatiotemporal distribution of microglia subtypes changes during disease progression by interacting with themselves and other cell types.

To conclude, the spatial statistics approach described herein addresses some of the current needs of modern biological research, by providing a means to fully explore the quantity and complexity of information contained in microscopic images^35, 36^. Our method is efficient, objective, and sensitive, and helps to reveal subtleties in microglia behaviour that are otherwise undetectable to the human eye. Importantly, the workflow described here comes with very few prerequisites and can therefore be considered a generic approach applicable to the entire CNS. Virtually any tissue of any species in any pathophysiological state can be assessed, as long as microglia are labelled with a sufficient signal-to-noise ratio. The current approach may thus also be applied to tissues stained with alternative microglia/macrophage markers (e.g. CD11b, isolection B4, F4/80) or with transgenically labelled cells in reporter lines (e.g. CX3CR1-GFP mice, *Tg*(*coro1a:eGFP; lyz:Dsred*) zebrafish)^17, 22, 37^, and aid to the maximization of information extracted from any study looking into microglia activation. As such, besides dramatically improving the quality of the data, with this microscopic spatial statistics approach we also react to the increasing scrutiny of the scientific, ethical and economic use of procedures involving animals.

## Methods

### Animals

All studies were conducted in compliance with the ARVO Statement for the Use of Animals in Ophthalmic and Vision Research and the European Communities Council Directive of 22 September 2010 (2010/63/EU) and the Belgian legislation (KB of 29 May 2013), and were approved by the KU Leuven institutional ethical committee (P025-2013). Wild type mice (C57Bl6/J; Charles River Laboratories), aged 2 to 4 months, were kept under a 12/12 h light-dark cycle and had *ad libitum* access to food and water.

### Surgical procedures

Intraorbital optic nerve crush (ONC) was performed as described^15^ in 14 animals. Briefly, a temporal incision was made in the conjunctiva and the exposed optic nerve was then crushed 1 mm from the globe with a cross-action forceps (no. 11262-30, Fine Science Tools) for 5 seconds. Funduscopy was performed before and after the procedure to assess integrity of retinal perfusion. Animals were sacrificed at 4 days post ONC.

All surgical procedures were performed under general anaesthesia with xylazine (i.p. 10 mg/kg body weight, XYL-M) and ketamine (i.p. 70 mg/kg body weight, Nimatek). After the procedure, an antibiotic ointment (tobramycin 3 mg/g, Tobrex) was applied to avoid infection and corneal desiccation. Naive eyes from a separate cohort of untreated animals served as controls (N = 14).

### Immunohistochemistry for Iba-1 on retinal whole-mounts

In order to label microglia (and potential macrophages infiltration from the circulation), an Iba-1 immunostaining was performed on retinal whole-mounts^30^. Mice were deeply anaesthetized (i.p. 30 mg/kg sodium pentobarbital, Nembutal) and sacrificed by cervical dislocation. Eyes were dissected and fixed for 1 hour in 4% PFA. Next, retinas were whole-mounted and again fixed for 1 hour in 4% PFA. Whole-mounted retinas were frozen for 15 minutes at −80 °C and rabbit anti-Iba-1 (Wako Chemicals, #019-19741) (1:1000), diluted in 10 mM phosphate-buffered saline (PBS) containing 2% Triton X-100 and 2% goat pre-immune serum, was applied overnight. The next day, a secondary goat anti-rabbit IgG antibody conjugated to an Alexa fluorophore-488 (Life Technologies) (1:200) was applied for 2 hours. Retinal whole-mounts were rinsed with PBS with 0.5% Triton X-100 in between steps, and mounted using mowiol mounting medium (10% mowiol 4–88, 40% glycerol, 0.1% 1,4-diazabicyclo-[2,2,2]-octane in 0.2 M Tris-HCl [pH 8.5]). Mosaic z-stack images (step size 3 μm; 20–30 stacks per image, comprising the retinal nerve fibre layer till the photoreceptor layer; 20x objective; 1 pixel = 1.24 μm) of the entire whole-mount were taken with a laser confocal scanning microscope (FV1000, Olympus), controlled with FluoViewer 4.2 software (Olympus), and a maximum intensity projection was made for further analysis.

### Automatic quantification of Iba-1 labelled microglia in retinal whole-mounts

(Step 1) Image analysis was performed using Fiji (ImageJ, v1.51 h, 64 bit Windows) software^38^ with MorphoLibJ (v1.3.1) integrated library and plugin^39^. Quantification of Iba-1 labelled microglia in retinal whole-mounts was achieved using a multi-step algorithm. First, segmentation of Iba-1 labelled microglia was achieved by applying a grey scale attribute opening filter (area minimum: 25 pixels; connectivity: 8) to an 8-bit maximum projection of the retinal whole-mount. An opening morphological filter (1-pixel radius octagon) was then used effectively to separate microglia soma from processes, before a maximum entropy threshold was used to segment microglia soma from image background.

(Step 2) Quantification of microglia soma was completed using the Fiji (ImageJ) Analyse Particles function with a particle size threshold of 10 pixels, to exclude small pixel noise and extract information regarding microglial soma area, centre of mass and roundness (equation 1).

1 $$Roundness=\frac{4A}{\pi {M}^{2}}$$

where A is the area of the microglia soma and M is the length of the major axis, derived from the longest axis of an ellipse fit to each microglia soma. In order to plot the above measures as a function of the distance from the ONH, the Euclidean distance of each microglia centroid, relative to the centre of the optic nerve head (ONH), was determined using a technique previously described for retinal ganglion cells (equation 2)^40^.

2 $$d=\sqrt{{(x-a)}^{2}+{(y-b)}^{2}}$$

where d is the microglia Euclidian distance, (a, b) is the coordinate centre of the ONH (determined manually) and (x, y) is the centroid of each microglia within a retina. Results were presented as percentage frequency distributions (bin size: 50 µm) to allow averaging of multiple retinas and comparisons between treatment groups.

(Step 3) Nearest neighbour distance (NND) was determined using a script for Fiji (ImageJ) developed by Y. Mao^41^ and only particles with a NND < 14 pixels were considered to be microglia, in order to exclude large process artefacts from subsequent analysis. Regularity index (RI) was calculated using equation 3:

3 $$RI=\,\frac{{X}_{NND}}{{\delta }_{NND}}$$

where X_NND_ is the average NND of a population and δ_NND_ is the standard deviation of that population^42^.

(Step 4) Whole-mount retinal area was determined as described previously^40^. Briefly, a low intensity threshold (0–5) was applied to each 8-bit image by operators masked to treatment groups. The pixel area of this selection was measured using Fiji (ImageJ) and converted to mm^2^, and from this selection the global microglia density was determined by dividing the total microglia population by total retinal area. The boundary of each retinal area were exported as a list of xy-coordinates and used in conjunction with microglia centroid coordinates to generate spatial point processes with retinal area boundaries.

A detailed description of the microglia segmentation process, as well as a link to the Fiji source code, is included as a supplementary file. The Fiji source code is also publicly available via http://bio.kuleuven.be/df/lm/research/methods. The data sets generated and analysed during the current study are available from the corresponding author on reasonable request.

### Parametric statistical analyses

For parametric statistics, normal distribution and parallel equal variance between groups was tested via D’Agostino-Pearson (omnibus K2) and Brown-Forsythe tests, respectively. A significance level of α < 0.05 was considered significant (*P < 0.05, **P < 0.01, ***P < 0.005). All data are described as mean ± SEM in the text. GraphPad Prism 5 (GraphPad Software) was used for all analyses, including Bland-Altman plots.

### *K*-means clustering and colour mapping

Soma area and roundness data for each microglia identified in this study were pooled before normalization with 90% winsorizing of each population to remove extreme values^43^. *K*-means clustering, using the protocol described by Hartigan and Wong^44^, was determined using the *K*-means function in the R stats software package (v3.3.1)^45^, with two centres corresponding to ‘low’ and ‘high’ activity microglia populations. After ascribing clusters, microglia populations were separated by retina, and colour maps of microglia centroids (x, y coordinates) plotted with ‘low’ activity microglia were identified as black and ‘high’ activity microglia in red.

### Ripley’s *K-* and *L*-functions

Microglia centroids from each retina were used in conjunction with ‘low’ or ‘high’ activity designations to create spatial point processes bounded by retinal area. To determine the extent of microglia distribution deviation from spatial homogeneity, Ripley’s *K*-functions^46^ were applied using the Kest function from the R spatstat software package^47^, to compare microglia spatial point patterns and assess deviation from compete spatial randomness. Initially, *K*-functions for unmarked spatial point processes (equation 4) were drawn as previously described^48^.

4 $$K(r)=\frac{1}{n}\sum _{i=1}^{n}{N}_{Pi}(r){\lambda }^{-1}$$

where N is the number of cells within distance *r* of another point, λ is the average cell density per unit area and *P*_*i*_ is the *i*th cell and the sum taken over n cells.

Compete spatial randomness was estimated using a random Poisson distribution *K*(*r*) = π*r* ^2^ where *r* > 0. Deviations from this estimation are indicative of incidence of clustering or dispersion. To facilitate data analysis, the variance stabilized Ripley’s *K*-function *L*(*r*) was calculated using the protocol proposed by Besag^49^ (equation 5).

5 $$L(r)={(K(r)/\pi )}^{0.5}$$

Using this transformation, under compete spatial randomness, *r* is linear and *L*(*r*) > *r* is indicative of clustering and *L*(*r*) < *r* is indicative of dispersal. *L*(*r*) can be further normalized to produce the *H*-function^50^, such that the expected value of *r* under compete spatial randomness is zero (equation 6).

6 $$H(r)=L(r)-r$$

This transformation is useful for the analysis of microglia spatial point processes. Using an adaptation of the work of Kiskowski *et al*.^48^, the radii of microglia territories (exclusion radii) can be estimated as the second minima – as microglia distribution is a dispersal rather than clustering process – of *H*(*r*), excluding the first minima that are an artefact of the algorithms NND exclusion radii. To determine whether the exclusion radii of ‘low activity’ (*Kii*), ‘high activity’ (*Kjj*) and intergroup (*Kij*) microglia populations changed upon ONC injury, changes in the spatial behaviour of ‘high’ and ‘low activity’ microglia populations across marked point processes were assessed, using the self-*K* and cross-*K* functions of the R spatstat software package for each retina.

### Dixon’s χ^2^-test

As microglia population clustering could occur independent of the existence of exclusion radii previously identified, a Dixon χ^2^-test was employed to evaluate spatial segregation of ‘low activity’ and ‘high activity’ microglia^51^. Using the dixon software package in R^52^, nearest neighbour contingency tables were constructed for microglia populations for each retina and results summed across multiple retina (Naive, ONC and Co-eye treatment groups) to produce a process-wide test statistics. Deviation from random distribution of microglia populations was assessed using an overall test of segregation between (C) and within (*z*_*ii*_ and *z*_*jj*_) groups, compared to results expected under random labelling, according to the methods described by Dixon *et al*.^51^.

## Electronic supplementary material

Supplementary data
