# Supplementary material for: Characterizing microglia activation: a spatial statistics approach to maximize information extraction
Source: Sci Rep. 2017 May 8;7:1576. doi: 10.1038/s41598-017-01747-8 (PMC5431479; doi:10.1038/s41598-017-01747-8)
Supplement: Supplementary file 1 — Supplementary data [file 41598_2017_1747_MOESM1_ESM.pdf]

Characterizing microglia activation: a spatial statistics approach to maximize information extraction.

Benjamin M. Davis, Manual Salinas-Navarro, M. Francesca Cordeiro, Lieve Moons, Lies De Groef

Supplementary Table 1. Overview of all Dixon's χ^2-test results across retinas from naive eyes, with N_i 'low activity' microglia and N_j 'high activity' microglia.

	N_i	N_j	C	df	p-value	z_{ii}	df	p-value	z_{jj}	df	p-value
#1	1965	1386	1.09	2	0.5788	0.9O	1	0.3667	0.89	1	0.3731
#2	1846	999	5.13	2	0.0769	-0.92	1	0.3600	-2.26	1	0.0236
#3	1437	666	1.69	2	0.4295	-0.10	1	0.9207	-1.22	1	0.2230
#4	1961	1065	5.11	2	0.0776	-2.00	1	0.0470	-1.85	1	0.0646
#5	2077	908	3.84	2	0.1463	-1.96	1	0.0500	-0.87	1	0.3818
#6	1811	1409	11.4	2	0.0033	0.08	1	0.9381	-3.01	1	0.0027
#7	1871	1010	4.67	2	0.0970	-2.15	1	0.0319	-1.16	1	0.2481
#8	1795	998	0.80	2	0.6709	0.43	1	0.6652	-0.49	1	0.6232
#9	1851	990	2.40	2	0.3005	1.49	1	0.1363	1.02	1	0.3093
#10	1679	700	3.68	2	0.1588	1.91	1	0.0560	0.34	1	0.4158
#11	1587	720	2.77	2	0.2506	-1.37	1	0.1692	-1.44	1	0.1509
#12	1926	744	3.24	2	0.1982	-1.03	1	0.3043	0.91	1	0.3647
#13	1745	967	0.40	2	0.8175	-0.53	1	0.5949	0.09	1	0.9321
#14	1657	1151	1.59	2	0.4525	-1.26	1	0.2084	-0.52	1	0.6037
	N_i	N_j	$\sum C$	df	p-value	$\sum z_{ii}^2$	df	p-value	$\sum z_{jj}^2$	df	p-value
ALL	25208	13713	47.81	28	0.0112	25.00	14	0.0346	26.55	14	0.0220

Supplementary Table 2. Overview of all Dixon's χ^2-test results across retinas from ONC eyes, with N_i 'low activity' microglia and N_j 'high activity' microglia.

	N_i	N_j	C	df	p-value	z_{ii}	df	p-value	z_{jj}	df	p-value
#1	1426	1908	13.84	2	0.0010	0.45	1	0.6503	-3.20	1	0.0014
#2	1968	2363	23.22	2	0.0001	-1.88	1	0.0604	-4.82	1	0.0001
#3	2190	1879	12.56	2	0.0019	2.19	1	0.0284	-1.60	1	0.1092
#4	1293	2074	20.78	2	0.0001	-3.09	1	0.0020	-4.35	1	0.0001
#5	1950	1869	0.70	2	0.7049	-0.66	1	0.5092	-0.75	1	0.4506
#6	2052	1615	5.59	2	0.0610	0.69	1	0.4931	-1.75	1	0.0808
#7	2018	2243	8.15	2	0.0170	1.60	1	0.1101	-1.35	1	0.1772
#8	1824	1936	1.00	2	0.6051	0.02	1	0.9855	-0.89	1	0.3752
#9	1844	2053	24.68	2	0.0001	-0.10	1	0.9203	-4.52	1	0.0001
#10	1446	2016	0.52	2	0.7703	-0.62	1	0.5369	-0.61	1	0.5441
#11	1414	1890	22.17	2	0.0001	0.64	1	0.5202	-3.89	1	0.0001
#12	1555	1966	6.62	2	0.0365	0.02	1	0.9812	-2.31	1	0.0211
#13	1434	1167	1.56	2	0.4582	0.85	1	0.3930	-0.45	1	0.6494
#14	1828	652	2.18	2	0.3357	-0.80	1	0.4220	-1.46	1	0.0891
	N_i	N_j	$\sum C$	df	p-value	$\sum z_{ii}^2$	df	p-value	$\sum z_{jj}^2$	df	p-value
ALL	24242	25631	143.57	28	0.00001	23.72	14	0.0495	104.80	14	0.00001

Supplementary Table 3. Overview of all Dixon's χ^2-test results across retinas from contralateral eyes, with N_i 'low activity' microglia and N_j 'high activity' microglia.

	N_i	N_j	C	df	p-value	z_{ii}	df	p-value	z_{jj}	df	p-value
#1	1301	625	3.88	2	0.1434	-1.85	1	0.0643	-0.12	1	0.9045
#2	1640	707	0.01	2	0.9931	-0.10	1	0.9214	0.02	1	0.9866
#3	2050	1015	1.48	2	0.4781	-0.43	1	0.6679	0.82	1	0.4110
#4	1482	887	1.16	2	0.5593	-1.01	1	0.3147	-0.05	1	0.9567
#5	2074	796	11.49	2	0.0032	-2.10	1	0.0355	1.52	1	0.1277
#6	2202	1084	2.07	2	0.3549	-1.01	1	0.3140	0.43	1	0.6657
#7	2275	1116	6.05	2	0.0486	-2.45	1	0.0143	-1.34	1	0.1806
#8	2175	861	12.01	2	0.0025	0.00	1	0.9989	-3.12	1	0.0018
#9	1984	961	0.65	2	0.7213	0.78	1	0.4374	0.16	1	0.8714
#10	1769	655	0.11	2	0.9442	0.01	1	0.9908	0.32	1	0.7508
#11	2072	764	0.66	2	0.7181	0.81	1	0.4165	0.31	1	0.7598
#12	1836	608	1.28	2	0.5267	-1.04	1	0.2974	-0.01	1	0.9958
#13	1520	615	8.48	2	0.0144	-2.52	1	0.0118	0.39	1	0.6966
#14	1301	625	3.88	2	0.1434	-1.85	1	0.0643	-0.12	1	0.9045
	N_i	N_j	$\sum C$	df	p-value	$\sum z_{ii}^2$	df	p-value	$\sum z_{jj}^2$	df	p-value
ALL	24380	10694	49.33	26	0.0038	24.77	13	0.0248	15.09	13	0.3017

Supplementary file: step-by-step protocol for automatic quantification of Iba-1 labelled microglia in retinal whole-mounts

The source code of the microglia anlysis script is publically available via http://bio.kuleuven.be/df/lm/research/methods

<u>(Step 1) Identification of Iba-1 labelled microglia</u>

- *Rationale:* Identification of Iba-1 labelled microglia in retinal whole-mounts was achieved using a five-step algorithm.
- *Protocol details:*
    1. Images (z-stacks, .lsm format) were acquired using 15% overlap in X and Y dimensions to permit automated stitching of the resulting images to a mosaic, using FluoViewer (v. 4.0) software (Olympus).
    2. .lsm files were imported into the Fiji programme, followed by conversion to 8-bit maximum projection images of the retinal whole-mount. This was done to reduce the size of the image file and reduce the dataset to two dimensions.
    3. Segmentation of bright Iba-1 labelled microglia was achieved using a grey scale attribute opening filter (area minimum: 25 pixels; connectivity: 8) from the MorphoLibJ package. These settings effectively isolated Iba-1 microglia from the dark background.
    4. Isolation of microglia soma (large round objects) from processes (long, thin objects) was achieved using an opening morphological filter (1-pixel radius octagon) from the MorphoLibJ software package. This approach effectively removed the majority of microglia processes from the image, but did sometimes struggle with large processes and at sites where processes intersected. A *post hoc* particle cut-off was used to overcome this problem (*crf.* step 3 below).
    5. Finally, microglia soma were segmented from image background using a maximum entropy threshold. This technique is preferable to Otsu and Li thresholding, which are more conservative in soma identification and led to undercounting.

(Step 2) Quantification of microglia somata

- *Rationale:* Once microglia soma had been successfully segmented from the image, the Fiji (ImageJ) Analyse Particles function was used to identify microglia soma from remaining small pixel noise and extract information regarding microglial soma area, centre of mass and roundness.
- *Protocol details:*
  1. A minimum particle size of 10 pixels (particle size threshold) was defined to exclude small pixel noise. This approach was found to be preferable over other noise reduction approaches (e.g., median/mean filtering) as the latter had a tendency to introduce additional particle artefacts into the image.
  2. Calculation of soma area and centre of mass.
  3. Calculation of soma roundness: $Roundness = \frac{4A}{\pi M^2}$

     where A is the area of the microglia soma and M is the length of the major axis, derived from the longest axis of an ellipse fit to each microglia soma.

  4. Calculation of the Euclidean distance of each microglia centroid, relative to the centre of the optic nerve head (ONH): $d = \sqrt{(x\text{-}a)^2 + (y\text{-}b)^2}$

     where d is the microglia Euclidian distance, (a,b) is the coordinate centre of the ONH (determined manually) and (x,y) is the centroid of each microglia within a retina.

  5. A data set comprising (x,y) microglia coordinates and the position (a,b) of the ONH for each whole-mounted retina was extracted from the Fiji script, and was used in conjunction with R spatstat software for subsequent analysis to allow averaging of multiple retinas and comparisons of microglia distribution between treatment groups.

(Step 3) Evaluation of microglia distribution

- *Rationale:* Microglia population distribution was characterized by nearest neighbour distance (NND) and regularity index, using a Fiji plugin 'NND' developed by Y. Mao [1].

- *Protocol details:*
    1. In order to exclude small particle artefacts that were being erroneously defined as microglia – these artefacts were determined to be large microglia processes that the script was incorrectly counting as soma –, only particles with a NND > 14 pixels were considered to be microglia.
    2. Calculation of regularity index: $RI = \frac{x_{NND}}{\delta_{NND}}$

    where X_{NND} is the average NND of a population and δ_{NND} is the standard deviation of the NND of that population.

(Step 4) Determination of the whole-mount retinal area

- *Rationale:* The boundaries of each retinal whole-mount were outlined and its area was measured, in order to calculate microglia densities and generate (x,y) coordinates for subsequent spatial statistics analysis.
- *Protocol details:*
    1. A low-intensity threshold (0-5) was applied to each 8-bit image.
    2. The pixel area of this selection was measured using Fiji and converted to mm^2. The global microglia density was determined by dividing the total microglia population by the total retinal area.
    3. The boundary of each retinal area was also exported as a list of (x,y) coordinates and used in conjunction with microglia centroid coordinates to generate spatial point processes with retinal area boundaries for subsequent Ripley's *K* analysis in the R spatstat software package.

Reference

1. Mao, Y. *Nearest Neighbor Distances Calculation with ImageJ*. 2016 [12/12/2016]; Available from: https://icme.hpc.msstate.edu/mediawiki/index.php/Nearest_Neighbor_Distances_Calculation_with_ImageJ.
